# Alveolar Ridge Bone Augmentation Using Carbonate Apatite Granules as a Novel Bone Substitute for Orthodontic Tooth Movement: A Report of the First Case

**DOI:** 10.7759/cureus.91521

**Published:** 2025-09-03

**Authors:** Kumiko Kamada, Atsushi Uesugi, Naoyuki Fukuda, Natsumi Takamaru, Naito Kurio

**Affiliations:** 1 Oral Surgery, Tokushima University, Tokushima, JPN

**Keywords:** guided bone regeneration, human bone apatite, low-crystalline carbonate apatite, novel bone graft substitute, orthodontic tooth movement (otm)

## Abstract

Orthodontic tooth movement (OTM) into severe alveolar bone defects is challenging, and alveolar bone augmentation is often required before OTM. However, few reports have been published regarding the augmentation with bone substitutes for OTM other than autologous bone. This report demonstrates that OTM into the previously augmented bone area with novel carbonate apatite (CO_3_Ap) granules as a novel bone substitute is possible and safe. Six months after bone augmentation with CO_3_Ap granules in the area of a severe alveolar bone defect, we confirmed sufficient new bone formation. Then, OTM into the augmented area was started. Clinical follow-up showed favorable tooth movement into the augmented area. Radiographic examination confirmed that the grafted granules were resorbed and replaced by new bone as the teeth moved, and adequate bone volume was present around the root after tooth movement. No abnormal root resorption was observed. No abnormal gingiva around the moved tooth was observed. The periodontal ligament space was maintained, and bony adhesion was not observed. OTM into the previously augmented bone area with CO_3_Ap granules was clinically successful. Bone augmentation with CO_3_Ap granules in severe alveolar bone defects is considered to be a safe and effective method for the subsequent OTM.

## Introduction

Sufficient alveolar bone is necessary to achieve the desired results in orthodontic tooth movement (OTM). Moving teeth into the severe alveolar bone defect areas can lead to periodontal disease or tooth loss. Therefore, alveolar bone augmentation is often necessary for OTM. Clinical studies have reported that alveolar bone augmentation is frequently required in orthodontic cases involving ankylosed or traumatized teeth, as well as in patients with congenital defects, highlighting the importance of effective grafting strategies in daily practice. Autologous bone is the gold standard as a grafting material for bone augmentation, and good results of OTM into the augmented bone area have been reported [1]. Although autologous bone is ideal, it has some restrictions, such as the need for additional donor site surgery or the limited amount of bone that can be harvested. Allografts from another human bone and xenografts from bovine bone are similar in structure and composition to autologous bone. But using these grafts has the risk of transmission of viruses or prions. Bone substitutes such as hydroxyapatite (HAp) and β-tricalcium phosphate (β-TCP) have excellent biocompatibility and are sometimes used for bone augmentation before OTM [2], but HAp is a calcium phosphate ceramic that remains rigid in the body for a long time, causing adverse effects such as tooth stagnation or severe root resorption. β-TCP dissolves rapidly, often before new bone has fully formed. Carbonate apatite (CO_3_Ap) naturally contains carbonate ions, which play a crucial role in bone remodeling and make its composition closer to that of living bone than HAp or β-TCP. So, an ideal bone substitute should be a CO_3_Ap. Recently, we successfully synthesized low-crystalline CO_3_Ap granules from calcium hydrogen phosphate dihydrate (DCPD) through a dissolution-precipitation reaction [3]. We have previously reported that the CO_3_Ap granules can restore alveolar bone defects in humans with sufficient bone volume and that the grafted CO_3_Ap granules are resorbed and replaced by living bone [4,5]. Therefore, the CO_3_Ap granules are considered to be a suitable bone substitute for bone augmentation. However, despite promising preclinical and clinical findings on the use of CO₃Ap for bone augmentation, no evidence has been reported regarding the safety and feasibility of OTM into CO₃Ap-augmented regions. Addressing this knowledge gap, we present here the first clinical case demonstrating successful OTM into an area augmented with CO₃Ap granules.

## Case presentation

**Figure 1:**
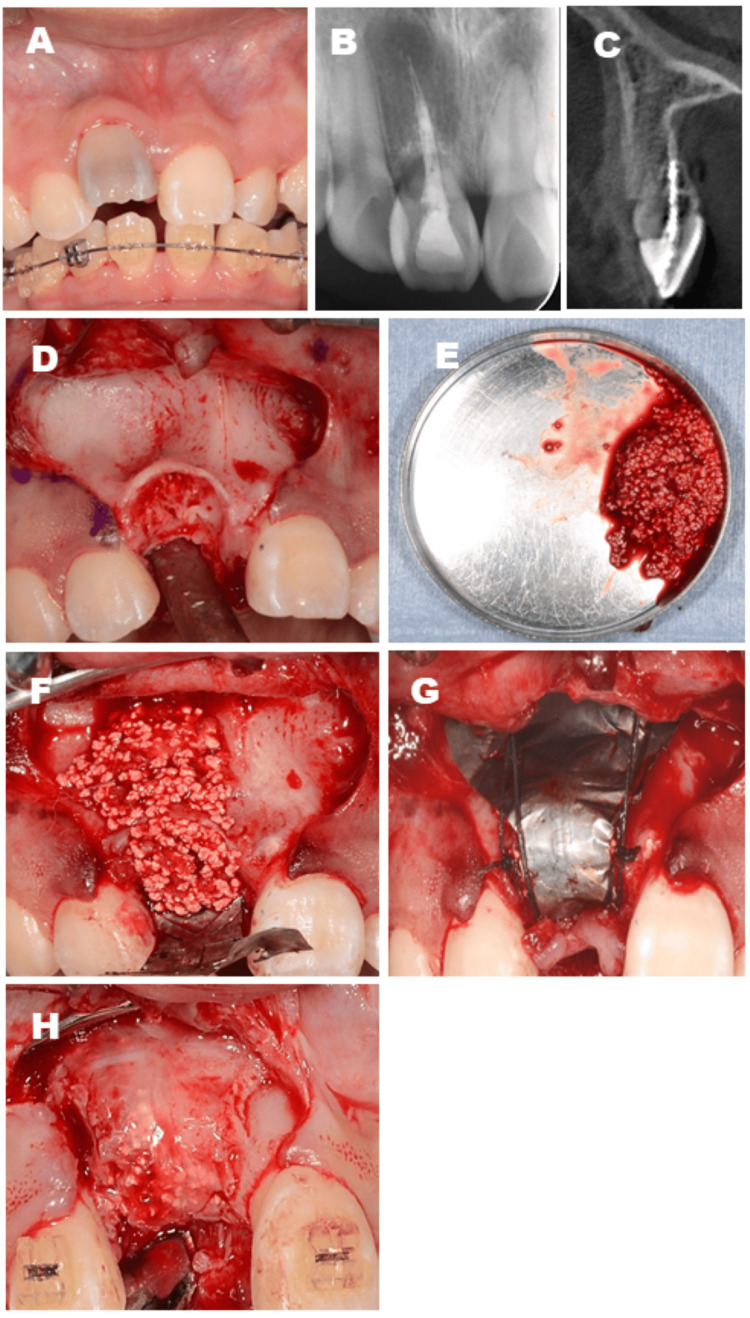
Grafting with carbonate apatite (CO₃Ap) granules for an alveolar bone defect after extraction of an ankylosed maxillary incisor. Preoperative intraoral and radiographic images. (A, B, C) Bony adhesion of the right maxillary central incisor was observed. Intraoperative images: (D) Severe bone defect after tooth extraction. (E, F, G) CO₃Ap granules mixed with venous blood were grafted onto the extraction site and covered with a titanium membrane. Intraoperative image of the secondary operation: (H) Sufficient bone augmentation was confirmed.

**Figure 2:**
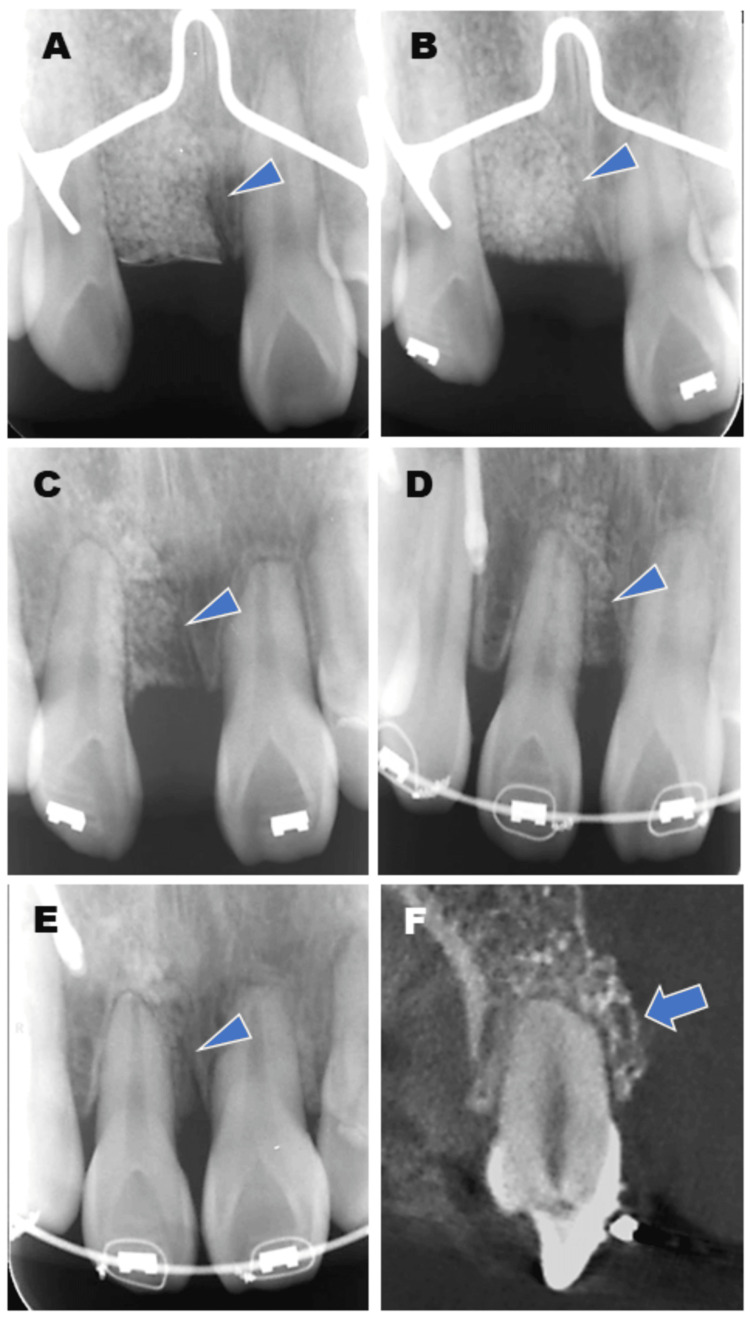
Time course observation of tooth movement by radiographs. (A) Dental X-ray image immediately after bone grafting with CO₃Ap granules. (B) Six months after the bone grafting, the border between the granules and the existing bone is becoming unclear. Orthodontic tooth movement (OTM) was started. (C) Two years after OTM, although small amounts of granules remain, alveolar bone with a well-defined structure is observed around the moving tooth. (D) Two years and eight months after OTM, tooth movement into the augmented area was completed. No abnormal findings were observed around the root of the tooth. (E, F) Dental X-ray and CBCT images one year after the completion of OTM. Mature alveolar bone was observed around the root. Arrowheads indicate residual granules, showing their gradual resorption and replacement with newly formed bone, and also highlight areas of bone remodeling in response to orthodontic forces. Arrows show sufficient labial bone volume, which is important for ensuring the stability of the tooth after orthodontic movement. CO₃Ap, carbonate apatite; CBCT, cone-beam computed tomography

**Figure 3:**
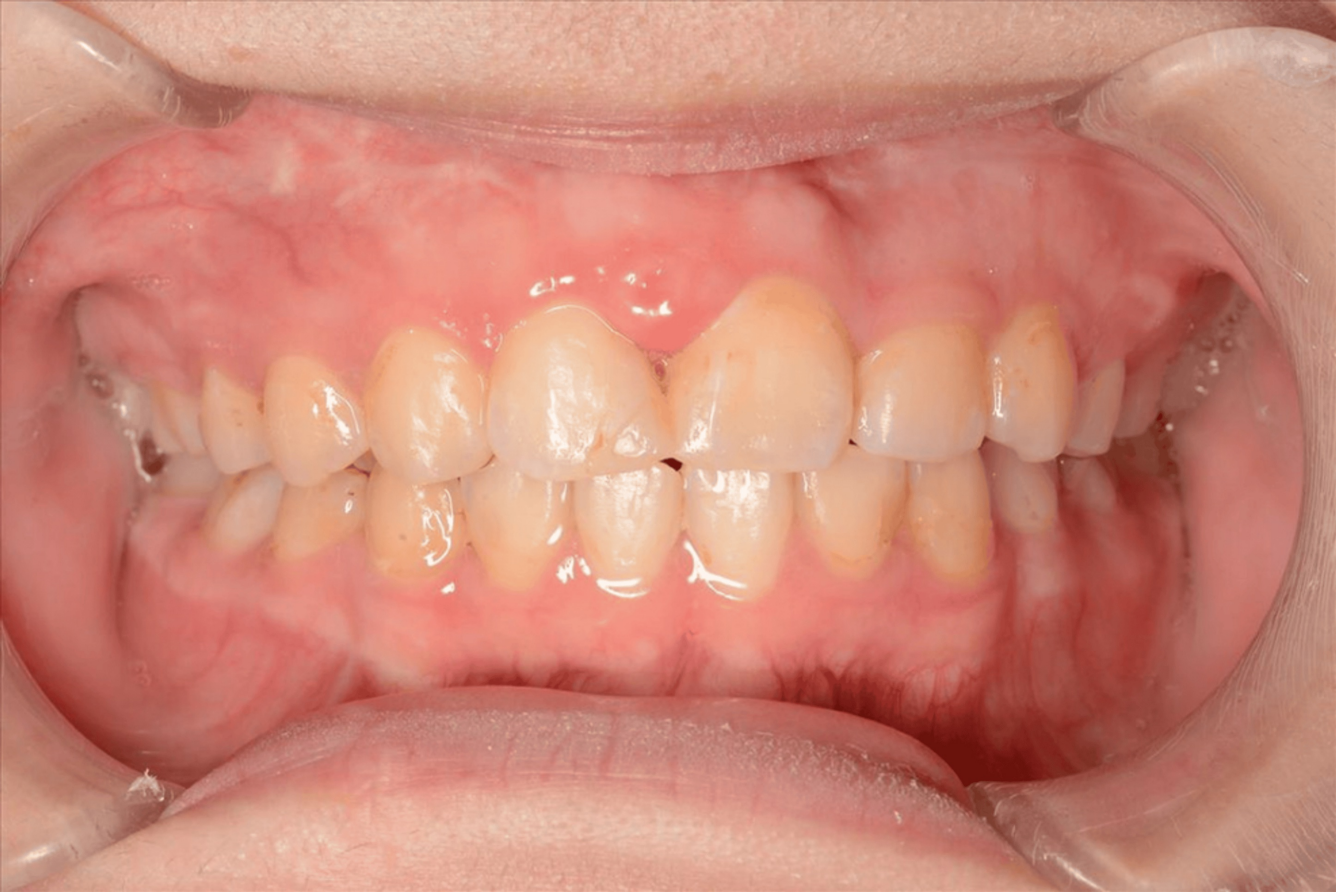
Intraoral photographs one year after completion of orthodontic treatment. Stable occlusion and periodontal conditions were maintained.

A 14-year-old girl visited our hospital, complaining of dentition malocclusion. The patient had a history of epilepsy and was receiving antiepileptic medication. At the initial examination, she presented with mild gingivitis, although her overall periodontal condition was otherwise within normal limits. The examination by the orthodontist revealed that she had an ankylosis in the right maxillary central incisor due to previous tooth trauma and had to have a tooth extraction for the orthodontic treatment (Figures 1A-1C). To close the tooth extraction space, OTM was planned. However, severe alveolar bone loss was predicted, and orthodontic treatment was expected to be difficult. Therefore, she was referred to our department for the tooth extraction and following bone augmentation. First, we proposed bone augmentation with autologous particulate cancellous bone marrow graft from the chin or iliac bone, but she disagreed with our plans due to the need for additional donor-site surgery. Therefore, we proposed another plan of bone augmentation with novel CO_3_Ap granules. The patient agreed with this plan. In January 2018, tooth extraction and bone augmentation were performed. After reflecting a mucoperiosteal flap in the gingiva, the right maxillary central incisor was extracted (Figure 1D). The graft bed was decorticated and filled with 1 g of the CO_3_Ap granules mixed with venous blood (Figures 1E, 1F). The grafted CO_3_Ap granules were covered with a microporous titanium membrane (Ti honeycomb membrane, Morita, Tokyo, Japan) (Figure 1G), and the operation was completed with suturing. Six months after the operation, a secondary operation was performed. After removing the membrane, a small amount of the granules remained on the surface of the augmented bone, but the granules in the deeper layer were mostly replaced by new bone (Figure 1H). Sufficient vertical and horizontal alveolar bone augmentation was confirmed, and then OTM was started by orthodontically moving the right lateral incisor. Two years and eight months later, the space of the augmented area was closed. No abnormal findings around the gingiva of the moved tooth were observed. X-ray findings revealed that the grafted CO_3_Ap granules were gradually absorbed and almost replaced with new bone (Figures 2A-2E). Root resorption was observed at one-fifth of the root tip; however, this was not abnormal resorption, which would have led to tooth extraction. The periodontal ligament space was also maintained, and no bony adhesion was observed (Figure 2F). Currently, one year has passed since the end of the tooth movement, and the clinical course is fruitful (Figure 3).

## Discussion

The ideal bone grafting material for bone augmentation for OTM is still human bone. Human bone apatite is not HAp but CO_3_Ap, which contains 6%-9% CO_3_^2-^. This CO_3_^2-^ affects osteoclastic bone resorption and promotes bone remodeling [6]. Therefore, an ideal bone substitute for bone augmentation should be a CO_3_Ap. However, this CO_3_^2-^ is readily decomposed by heat; it was difficult to synthesize an artificial CO_3_Ap block without sintering. Recently, we succeeded in artificially synthesizing low-crystalline CO_3_Ap granules from dicalcium phosphate dihydrate (DCPD) through the dissolution-precipitation reaction for the first time in the world [7]. Since this novel CO_3_Ap granules are not sintered and are transformed at low temperatures, the crystallinity is low and includes almost the same amount of CO_3_^2-^ as human bone. In our basic research, CO_3_Ap granules had superior osteogenic potential compared to other calcium phosphates such as HAp and β-TCP, and eventually resorbed and replaced by new bone, restoring alveolar bone defects [6].

**Table 1:** Clinical timeline summary of the present case. CO₃Ap, carbonate apatite

Time point	Clinical event	Key observations/outcomes
Baseline (first operation)	Extraction of ankylosed right maxillary central incisor and bone augmentation with CO₃Ap granules mixed with venous blood, covered by titanium membrane	Severe alveolar bone defect confirmed; graft placed successfully
6 months post-surgery (secondary operation)	Removal of the titanium membrane	Residual granules observed superficially; deeper granules mostly replaced with new bone; sufficient vertical and horizontal augmentation confirmed
6 months post-surgery (OTM initiation)	Start of orthodontic tooth movement (OTM)	OTM was initiated safely based on adequate new bone formation
2 years after OTM initiation	Clinical and radiographic follow-up	Ongoing space closure observed; radiographs showed gradual replacement of granules with new bone
2 years 8 months after OTM initiation	Completion of orthodontic movement into the augmented area	Space fully closed; no abnormal gingival findings; root resorption limited to apical tip, not clinically severe
1 year post-OTM completion	Long-term follow-up (dental X-ray and CBCT)	Mature alveolar bone present; periodontal ligament space maintained; no bony adhesion

In this case, we also confirmed sufficient alveolar bone augmentation by the CO_3_Ap granules, as we have previously reported [6]. Bone remodeling of the grafted CO_3_Ap granules was observed by X-ray examination. The grafted CO_3_Ap granules were gradually absorbed and replaced by new bone as the tooth moved. At the end of the OTM, new bone was observed around the moved tooth root, confirming the safe tooth movement through the augmented bone area. This clearly showed that the augmented bone by CO_3_Ap granule has bone remodeling ability for orthodontic force. Regarding the timing of orthodontic force application after bone augmentation, there is no consensus [8]. It has been reported that osteocytes in mature bone play an important role in OTM [9]. The application of orthodontic force to the teeth induces strong expression of the receptor activator of nuclear factor-kappa ligand β in osteocytes, which activates osteoclast differentiation and promotes alveolar bone remodeling, resulting in tooth movement. This suggests that some mature alveolar bone is required for tooth movement. Thus, we started orthodontic force application at six months after bone augmentation, referring to our studies that most of the CO_3_Ap granules were replaced with new bone in about six months [6]. In this case, the same degree of apical root resorption was observed on the upper anterior teeth. Root resorption during orthodontic treatment is commonly observed, with an estimated incidence of 6% to 13% and an average root resorption of 2.5 mm reported [10]. It can occur on all teeth, but it is likely to occur on the upper and lower anterior teeth. The reason for root resorption involves various factors such as the magnitude of orthodontic force, the type of force, the direction of tooth movement, and the duration of orthodontic treatment. Furthermore, patients’ status, such as gender, age, type of occlusion, morphology of the root, and dental history of tooth trauma, can also be a risk factor for root resorption. In our case, the patient has a history of dental trauma and requires a long distance of tooth movement of the lateral incisor. These may contribute to the root tip resorption. Therefore, the root resorption in the present case was comparable to that which occurs with conventional orthodontic treatment and was not considered abnormal. Although detailed quantitative measurements such as volumetric CBCT analysis, exact bone gain were not available for this case, we carefully documented clinical and radiographic findings to evaluate treatment outcomes. Future studies should incorporate standardized quantitative indices to enable broader comparison and reproducibility across cases. Overall, OTM to the augmented bone area by the CO_3_Ap granules was successful and clinically acceptable.

## Conclusions

This case report demonstrates that alveolar bone augmentation using CO₃Ap granules enabled safe OTM through the augmented area, with favorable bone remodeling and maintenance of periodontal health. These findings suggest that CO₃Ap granules may serve as a useful bone substitute in orthodontic treatment. However, as this is a single case, the broader clinical applicability remains preliminary. Further large-scale and long-term studies are needed to validate the safety and efficacy of CO₃Ap in orthodontic and other clinical applications.
